# Variations in medicare reimbursements among surgical oncologists who are US versus international medical graduates

**DOI:** 10.1002/wjs.12458

**Published:** 2024-12-22

**Authors:** Muhammad Muntazir Mehdi Khan, Abdullah Altaf, Mujtaba Khalil, Sidharth Iyer, Razeen Thamachack, Abdul Hadi Shahid, Zayed Rashid, Timothy M. Pawlik

**Affiliations:** ^1^ Department of Surgery The Ohio State University Wexner Medical Center and James Comprehensive Cancer Center Columbus OH USA; ^2^ Medical College Aga Khan University Hospital Karachi Pakistan

**Keywords:** biliary, colorectal, gastrointestinal, hepatic, liver, oncology, outcomes

## Abstract

**Introduction:**

We sought to assess the variations in practice metrics and billing practices among US Medical Graduates (USMGs) and International Medical Graduates (IMGs) in surgical oncology who serve a fee‐for‐service population.

**Methods:**

Medicaid Services Medicare fee‐for‐service provider utilization and payment files were used to obtain publicly available data between January 1, 2021, and December 31, 2021. Comparisons were conducted using the *t*‐test for parametric variables and Wilcoxon rank‐sum for nonparametric variables.

**Results:**

A total of 952 surgical oncologists (IMGs: *n* = 102 [10.7%]) were included in the analytic cohort. The average risk score among beneficiaries treated by IMGs was higher than USMGs (1.70 [0.04] vs. 1.46 [0.02], *p* < 0.001) and IMGs also had a higher total number of unique codes (47.0 [IQR: 36.0–69.0] vs. 38.0 [IQR: 24.0–60.0], *p* < 0.05). IMG surgical oncologists had higher payment‐per‐service amounts ($236.56 [10.34] vs. $196.20 [$2.65]; *p* < 0.05), charge‐per‐service amounts ($1242.48 [$83.14] vs. $1014.89 [$26.13]; *p* < 0.05), and higher total submitted charges ($400,373.26 [$342,978.45] vs. $360,020.29 [$523,675.91]; *p* < 0.05). IMGs provided a higher percentage of procedural services (34.1% vs. 27.9%; *p* < 0.001) and treatment services (2.1% vs. 1.9%; *p* < 0.001) versus USMGs. Female surgical oncologists, particularly female IMGS, billed lower annual mean Medicare charges (female IMGS: $295,383 vs. male IMGs: $424,407 vs. female USMGs: $294,168 vs. male USMGs: $414,543; *p* < 0.001).

**Conclusions:**

IMGs provided more procedural services, cared for patients with a higher average risk score, and performed a greater variety of procedures compared with USMGs. Consequently, IMGs had higher mean annual charges, payment‐per‐service, and charge‐per‐service amounts.

## Graphical abstract

Variations in Medicare Reimbursements among US and International Medical Graduates.

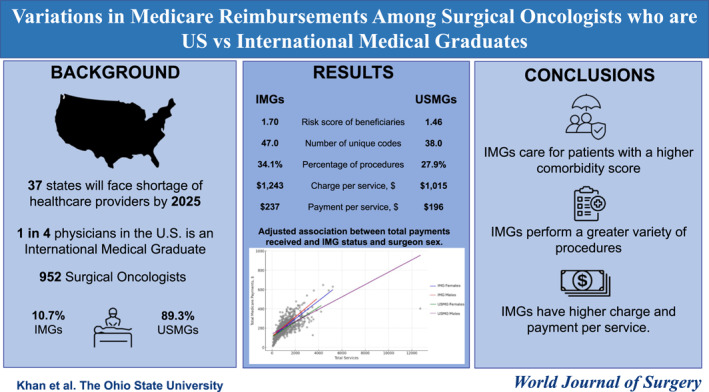

## 1 INTRODUCTION

Over the past several decades, the US healthcare workforce has undergone significant transformation with international medical graduates (IMGs)—defined as physicians who have received their medical education outside of the United States or Puerto Rico—becoming increasingly more common. IMGs have been instrumental in strengthening the US physician workforce and alleviating physician shortages to the extent that IMGs comprise nearly one out of every four medical doctors (25%) in the US^1^, ^2^, ^3^ Nearly 90% of IMGs are foreign‐born with over two‐thirds originating from low‐resource, developing countries.^3^ IMGs not only diversify the US healthcare workforce but have been reported to address the needs of broad patient populations, care for more vulnerable communities, and mitigate shortages in underserved areas.^4^, ^5^, ^6^, ^7^ According to the estimates of the Bureau of Health Workforce, 37 states in the US will face shortages of healthcare providers by 2025.^8^ As such, it is projected that IMGs will make up over 35% of primary care physicians in the US in the near future.

Despite making up a significant portion of the physician workforce and a growing workforce need in the US healthcare system, IMGs frequently encounter major challenges ranging from medical licensing and residency placement to employment opportunities and visa applications.^9^, ^10^, ^11^ These hurdles, compounded by institutional biases, impact their career trajectories and financial compensation.^3^, ^9^ Despite these systemic disadvantages and differences in practice patterns, IMGs have been reported to achieve comparable patient outcomes compared with US medical graduates (USMGs).^12^, ^13^, ^14^ In addition, IMGs often address the physician shortage, particularly in underserved and rural areas.^15^ Healthcare professionals in these underserved areas may have longer work hours and disparate financial compensation.^16^ Historically marginalized and underrepresented individuals may be subject to discriminatory wage gaps.^17^ To date, differences in practice patterns and payments among IMGs compared with their USMG counterparts have not been examined.

The overall income of a surgical oncologist can come from various sources; however, a significant portion is derived from fee‐for‐service reimbursement for the medical services provided.^18^ Understanding variations in Medicare reimbursements between USMGs and IMGs can provide insights into billing practices, disparities in compensation, patient demographics, and procedural workload.^17^, ^19^, ^20^ These findings can help optimize workforce utilization, especially in light of the growing disparities within the surgical workforce.^20^ Previous studies have examined the clinical outcomes, experiences, and challenges associated with IMGs in the US healthcare system; however, data on practice patterns, which procedures are most commonly performed, and compensation patterns for services delivered by IMGs versus USMGs are scarce.^3^, ^13^, ^14^, ^19^, ^21^, ^22^ Therefore, using the national Medicare service and payment data, the objective of the current study was to characterize variations in practice metrics and billing practices among IMGs and USMGs delivering surgical oncology care for a fee‐for‐service population. In addition, we sought to identify factors associated with potential differences in Medicare reimbursements among IMGs and USMGs.

## 2 METHODS

### 2.1 Data source and study population

The Centers for Medicare & Medicaid Services (CMS) Medicare fee‐for‐service provider utilization and payment files were queried to obtain publicly available data. The study year was restricted to January 1, 2021, and December 31, 2021 to eliminate any temporal biases.^23^ Information regarding individual surgeons, including demographic details such as geographic location, medical school attended, and surgeon sex, along with data on annual services provided to Medicare fee‐for‐service patients, was obtained from provider summary files sorted by national provider identifier (NPI). These data included the total submitted charges, payments received, unique services provided, and the number of beneficiaries served. Detailed data files, categorized by both NPI and current procedural terminology (CPT) codes, offered a detailed breakdown of a surgeon's yearly practice composition, the extent of services provided, and the corresponding payments received. Data that covered claims submitted for services related to traditional Medicare beneficiaries were included, while data on Medicare advantage patients were excluded.

Only surgeons with a primary specialty code for “surgical oncology” were included in the analytic cohort. Medical school of graduation and graduation year were obtained from the Doctors and Clinicians National Downloadable File and linked to the primary data sets through the NPI.^24^ Information on missing medical schools was completed using an online website search of the surgeons' respective hospitals and medical schools. All the data used in this study were publicly available and did not contain individual patient information. Surgeons who had 10 or fewer services were excluded from the analysis to safeguard patient privacy. Moreover, surgeons without an MD, DO, or equivalent credentials were excluded. The need for informed consent for deidentified data was waived by the institutional review board (IRB) of the Ohio State University. This study followed the Strengthening the Reporting of Observational Studies in Epidemiology reporting guidelines.

### 2.2 Primary exposures and outcome interest

The primary exposure of interest was the medical school of graduation (i.e., USMG or IMG). IMGs were defined as surgeons who attended medical school outside the US and Puerto Rico, whereas USMGs were defined as surgeons who attended medical school within the US and Puerto Rico. Variables such as the number and types of services provided, the unique billing codes utilized, and the number of beneficiaries attended to in the analytic cohort were collected. An average hierarchal condition category risk score was utilized for beneficiaries to account for comorbidities.^25^ Medicare's Hierarchical Condition Category system provides hierarchical risk scores, which adjust for patient comorbidities and demographics to estimate healthcare costs. Additionally, the Medicare dataset incorporates zip code‐level clustering to account for geographic disparities in healthcare access, ensuring consistent risk adjustment for valid comparisons across populations. Years in practice were estimated using medical school graduation year. The primary outcomes of interest were annual total submitted charges, payments, mean charge per service, and mean payment per service, as well as the payment‐to‐charge ratio. All outcomes were compared in 2021 US dollars.

### 2.3 Statistical analysis

Continuous variables were presented as median with interquartile range and mean with standard error (SE) for nonparametric (i.e., beneficiary, services, and CPT codes) and parametric (i.e., payments and charges) data, respectively. Univariable comparisons were conducted using the *t*‐test for parametric variables and Wilcoxon rank‐sum for nonparametric variables. Stratified multivariable linear regression, accounting for clustering at the zip code level, was employed to characterize the association of IMG status with standardized payments, number of services and beneficiaries, and average beneficiary risk score.

Further data analysis was conducted to examine variations in practice composition. The services rendered were classified based on the Restructured Berenson‐Eggers Type of Service, BETOS Classification System (RBCS), which categorizes procedure codes into clinically meaningful categories such as imaging and procedures.^20^, ^26^ To assess the distribution of these categories among IMGs and USMGs, the proportion of each category in relation to their total services was calculated and then compared using the Pearson χ2 test. A comparison of individual services between IMGs and USMGs was performed by matching CPT codes with the use of *t*‐test to detect any discrepancies. Additionally, a stratified analysis was performed to examine possible differences in male and female surgeons. All statistical analyses were derived from two‐tailed tests and a *p*‐value of <0.05 was considered statistically significant. The analyses were performed using STATA, version 18.0 (StataCorp, College Station, TX).

## 3 RESULTS

### 3.1 Baseline characteristics

A total of 952 surgical oncologists (IMGs: *n* = 102 [10.7%], USMGs: *n* = 850 [89.3%]) were included in the analytic cohort. Overall, the mean (SE) age of the beneficiaries was 72.6 (0.07) years, and the average risk score was 1.48 (0.02) for the entire cohort. USMGs were more likely to care for non‐Hispanic white beneficiaries (112.0 [IQR: 68.0–183.5] vs. 93.0 [IQR: 66.0–146.0], *p* < 0.05), but there was no difference in the treatment of male versus female, racial/ethnic minorities, or dual Medicare/Medicaid beneficiaries among IMGs versus USMGs. The average risk score among beneficiaries treated by IMGs was, however, higher than USMGs (1.70 [0.04] vs. 1.46 [0.02], *p* < 0.001). IMGs also had a higher median number of years in practice (22 [IQR: 18–30] vs. 20 [IQR: 14–29], *p* < 0.05) and more total number of unique codes submitted for payment (47.0 [IQR: 36.0–69.0] vs. 38.0 [IQR: 24.0–60.0], *p* < 0.05) compared with USMGs (Table 1).

**TABLE 1:** Medicare charges, payments, practice volume, and beneficiary metrics among practices among US Medical Graduates versus International Medical Graduates.

	IMG	USMG	All	
	(*N* = 102, 10.7%)	(*N* = 850, 89.3%)	(*N* = 952)	*p*‐value
Surgeon sex				**<0.001**
Female	19 (18.6%)	385 (45.3%)	404 (42.4%)	
Male	83 (81.4%)	465 (54.7%)	548 (57.6%)	
Charges and payments, mean (SE)				
Total submitted charges, $	400,373.26 (342,978.45)	360,020.29 (523,675.91)	364,343.83 (507,417.23)	**0.045**
Total payments, $	77,897.80 (5480.92)	72,631.27 (2637.13)	73,195.54 (2426.517)	0.092
Total standardized payments, $^a^	73,423.19 (5090.18)	68,998.41 (2495.77)	69,472.49 (2293.85)	0.108
Payment‐to‐charge ratio	0.23 (0.009)	0.23 (0.003)	0.23 (0.003)	0.510
Charge per service	1242.48 (83.14)	1014.89 (26.13)	1039.27 (25.06)	**0.005**
Payment per service	236.56 (10.34)	196.20 (2.65)	200.52 (2.65)	**<0.001**
Beneficiary characteristics, median (IQR)				
Average age, mean (SE)	72.47 (0.22)	72.55 (0.071)	72.54 (0.07)	0.718
Male, No.	49.5 (29.0–74.0)	49.0 (22.5–82.5)	49.0 (24.0–80.0)	0.693
Female, No.	62.5 (41.0–96.0)	64.0 (44.0–104.5)	64.0 (43.0–104.0)	0.435
Non‐Hispanic white, No.	93.0 (66.0–146.0)	112.0 (68.0–183.5)	110.0 (68.0–178.0)	**0.045**
Racial/ethnic minority, No.	27.5 (15.5–41.0)	26.0 (15.0–42.0)	26.0 (15.0–42.0)	0.962
Dual Medicare/Medicaid eligibility, No.	22.0 (16.0–34.0)	20.0 (14.0–30.0)	21.0 (15.0–30.0)	0.136
Average risk score, mean (SE)^b^	1.70 (0.04)	1.46 (0.02)	1.48 (0.02)	<**0.001**
Practice volume, median (IQR)				
Total services, No.	283.5 (178.0–464.0)	296.5 (183.0–496.0)	294.5 (182.0–488.5)	0.573
Total beneficiaries, No.	119.0 (83.0–175.0)	118.5 (80.0–192.0)	119.0 (80.5–186.5)	0.343
Unique codes, No.	47.0 (36.0–69.0)	38.0 (24.0–60.0)	39.0 (24.0–61.0)	**<0.001**
Years of practice	22 (18–30)	20 (14–29)	20 (14–29)	**0.009**

*Note*: Bold values represent statistical significance (*p* < 0.05). Statistics presented: mean (SE), median (interquartile range).

^a^ Standardized payments indicates adjustment for geographic differences.

^b^ Risk score indicates adjustment for different patient risk profiles.

### 3.2 Variations in charges, payments, and practice volume

To evaluate the association between IMG status and practice volume, the total number of annual services provided, beneficiaries treated, and unique service codes used were assessed. IMG surgical oncologists had a higher payment‐to‐service ratio ($236.56 [10.34] vs. $196.20 [$2.65]; *p* < 0.05), charge‐to‐service ratio ($1242.48 [$83.14] vs. $1014.89 [$26.13]; *p* < 0.05), and higher total submitted charges ($400,373.26 [$342,978.45] vs. $360,020.29 [$523,675.91]; *p* < 0.05). Total payments received were comparable between IMGs and USMGs.

### 3.3 Variations in practice composition

To further analyze practice composition based on average national Medicare allowance, unique services were stratified into four payment categories for each CPT code. A total of 280,654 services (IMGs: *n* = 25,243 [9.0%], USMGs: *n* = 255,411 [91.0%]) were included. Overall, the top‐paying services (fourth quartile) comprised a greater proportion of all services performed by IMGs (39.3% vs. 32.6%; *p* < 0.001) compared with USMGs. To examine the variety of services provided by IMGs and USMGs, individual surgeon Healthcare Common Procedure Coding System codes were aggregated using the Restructured BETOS Classification System taxonomy. Notably, IMGs provided a higher percentage of procedural services (34.1% vs. 27.9%; *p* < 0.001) and treatment services (2.1% vs. 1.9%; *p* < 0.001) versus USMGs. In contrast, USMGs provided a greater percentage of evaluation and management (E&M) services (65.9% vs..57.1%; *p* < 0.001) (Table 2).

**TABLE 2:** Types of services provided to Medicare fee‐for‐services beneficiaries among practices among US Medical Graduates versus International Medical Graduates.

	IMG	USMG	
Surgical oncologists	*N* = 102, 10.7%	*N* = 850, 89.3%	*p*‐value
Total services by payment group	**25,243 (9.0%)**	**255,411 (91.0%)**	**<0.001**
Q1	3832 (15.2%)	38,731 (15.2%)	
Q2	5325 (21.1%)	54,847 (21.5%)	
Q3	6165 (24.4%)	78,486 (30.8%)	
Q4	9.921 (39.3%)	83,347 (32.6%)	
Service codes by revised BETOS classification	**2887 (43.2%)**	**3797 (66.8%)**	**<0.001**
E&M	1649 (57.1%)	2504 (65.9%)	
Imaging	130 (4.5%)	123 (3.2%)	
Procedure	985 (34.1%)	1060 (27.9%)	
Test	62 (2.1%)	72 (1.9%)	
Treatment	61 (2.1%)	38 (1.0%)	

*Note*: Bold values represent statistical significance (*p* < 0.05).

Abbreviations: BETOS, Berenson‐Eggers Type of Service; E&M, Evaluation and Management.

### 3.4 Variation in billing and coding practices among IMGs and USMGs

The 10 most common procedures performed by IMG and USMG surgeons were analyzed based on total service counts in relation to the national average Medicare‐allowed amounts for these services. For each surgical subspecialty, the 10 leading procedures accounted for 74.7% of the total procedure volume for IMG and 73.0% for USMG surgeons, respectively. The Medicare‐allowed amounts for these procedures accounted for $3753.1 or $50.24 per 1% for IMG and $3947.0 or $54.07 per 1% for USMG surgical oncologists, respectively (Table 3).

**TABLE 3:** Top 10 procedural codes utilized by International Medical Graduate and practices among US Medical Graduate surgical oncologists according to the total number of services.

IMG			USMG		
CPT code (name)	n (%)	Allowed Medicare amount, $	CPT code (name)	n (%)	Allowed medicare amount, $
Surgical oncology procedures	2818			41,809	
Insertion of central venous catheter and implanted device for infusion beneath the skin, patient 5 years or older	385 (13.7%)	329.5	Partial removal of breast	8360 (20.0%)	673.7
Partial removal of breast	363 (12.9%)	636.3	Lymph node imaging during surgery	6828 (16.3%)	144.6
Fluoroscopic guidance for insertion, replacement, or removal of central venous access device	336 (11.9%)	19.0	Biopsy or removal of lymph nodes of under the arm, open procedure	6722 (16.1%)	276.0
Biopsy or removal of lymph nodes of under the arm, open procedure	258 (9.2%)	261.7	Insertion of central venous catheter and implanted device for infusion beneath the skin, patient 5 years or older	1932 (4.6%)	320.7
Lymph node imaging during surgery	221 (7.8%)	143.5	Fluoroscopic guidance for insertion, replacement, or removal of central venous access device	1791 (4.3%)	18.8
Diagnostic examination of voice box using flexible endoscope	206 (7.3%)	62.2	Removal of malignant growth (over 4.0 cm) of the trunk, arms, or legs	1199 (2.9%)	229.0
Placement of flap to repair the abdominal wall	116 (4.1%)	310.9	Total removal of breast	1036 (2.5%)	1148.7
Muscle flap wound repair at the trunk	89 (3.2%)	1067.8	Tissue transfer repair of the wound (30.0 sq centimeters)	1026 (2.5%)	223.6
Diagnostic examination of the anus using an endoscope	74 (2.6%)	14.2	Ultrasound guidance for accessing into the blood vessel	815 (2.0%)	13.9
Removal or exploration of parathyroid glands	57 (2.1%)	908.0	Removal or exploration of parathyroid glands	793 (1.9%)	898.1
Sum	2105 (74.7%)	3753.1	Sum	30,502 (73.0%)	3947.0

A matching of procedures using unique CPT codes was then performed considering the total number of beneficiaries and services, as well as the location in which the service was provided to allow for the comparison of mean charges submitted and payments received per service between IMG and USMG surgical oncologists. Notably, IMGs submitted higher charges (Difference: $233.24 [IMG: $2188.69, USMG: $1955.45; *p* < 0.05]) and received slightly lower payments (Difference: $31.78 [IMG: $363.23, USMG: $331.46; *p* < 0.05]) per service than their USMG colleagues. Ultrasound guidance for tissue destruction and partial removal of the pancreas, bile duct, and small bowel with connection of pancreas to small bowel were the procedures that contributed the most to the payment discrepancies (Supplementary Table 1).

**FIGURE 1:**
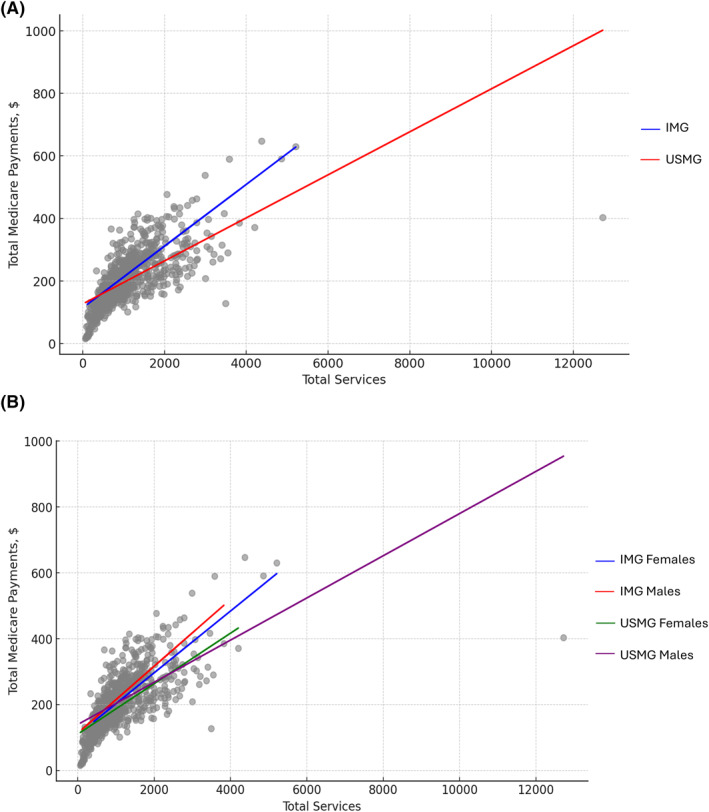
(A) adjusted association between total payments received and International Medical Graduate (IMG) status. (B) adjusted association between total payments received, IMG status, and surgeon sex.

On stratified analysis by surgeon sex, female surgical oncologists, particularly female IMGS, billed lower annual mean Medicare charges (Female IMGS: $295,383 vs. Male IMGs: $424,407 vs. Female USMGs: $294,168 vs. Male USMGs: $414,543) and received lower mean reimbursements (Female IMGS: $57,798.5 vs. Male IMGs: $82,498.8 vs. Female USMGs: $61,392.4 vs. Male USMGs: $81,936.5) (both *p* < 0.001). To evaluate the association between surgeon sex and practice volume, the total number of annual services provided, beneficiaries treated, and unique service codes used were assessed. Similar to charge and payment data, female surgical oncologists billed for fewer median total services (Female IMGS: 264 vs. Male IMGs: 311 vs. Female USMGs: 267 vs. Male USMGs: 323), had fewer beneficiaries (Female IMGS: 92 vs. Male IMGs: 119 vs. Female USMGs: 108 vs. Male USMGs: 128) and utilized fewer unique service codes (Female IMGS: 37 vs. Male IMGs: 54 vs. Female USMGs: 26 vs. Male USMGs: 50) (all *p* < 0.05). Moreover, the mean (SE) payment (Female IMGS: $231.4 vs. Male IMGs: $2377 vs. Female USMGs: $179.2 vs. Male USMGs: $210.3) and charge per service (Female IMGS: $1308.4 vs. Male IMGs: $1227.4 vs. Female USMGs: $902.1 vs. Male USMGs: $1108.2) were much lower for female than male surgical oncologists (both *p* < 0.001) (Supplementary Table 2) (Figure 1).

## 4 DISCUSSION

IMGs represent a quarter of the healthcare workforce in the US healthcare system and IMG providers contribute to a wide variety of medical subspecialties.^1^ Despite their vital role in filling healthcare workforce gaps and contribution to diversifying healthcare providers, IMGs continue to face obstacles in training and practice. These challenges include adapting to a new healthcare system, tackling visa issues, dealing with systemic biases, and adjusting to a new country.^9^, ^11^, ^27^, ^28^ Previous work by our group and others has demonstrated that immigrant doctors work longer hours, serve socially vulnerable populations, and work in areas with low provider density.^19^ Due to these reasons, differences in the practice patterns and financial compensation among IMGs versus USMGs may exist. To date, these differences have not been investigated among surgeons or surgical oncologists. Therefore, the current work was important as we specifically characterized variations in practice metrics and financial reimbursements among IMGs and USMGs using recent national Medicare service and payment data. Of note, IMGs had higher total submitted charges, payment‐per‐service, and charge‐per‐service amounts compared with USMGs; however, there were no differences in mean annual payments. Further analyses revealed that IMGs were more likely to provide procedural services compared with USMGs, whereas USMGs more often submitted E&M billing codes. Interestingly, there were also sex‐based differences among IMG and USMG surgical oncologists. For example, female IMGs were the group of surgeons with the lowest mean annual payments. To the best of our knowledge, the current study is the first study to evaluate variations in practice patterns and payment differences in charges and payments among IMGs and USMGs providing care for Medicare beneficiaries.

IMGs had higher total submitted charges, payment per service, and charge per service compared with USMGs. While reasons for the differences in charges and payments were likely multifactorial, there were likely several reasons for the noted disparities. For instance, IMGs are known to care for populations with high social vulnerability, as well as provide care in areas with low provider density.^19^ Moazzam et al. had demonstrated that immigrant doctors worked more hours annually compared with US born citizens.^19^ In a separate study, Glover et al. reported that IMG internists were more likely to care for Medicare patients with more complex medical needs than USMG internists.^29^ These variations in practice patterns may explain some of the differences in payments among IMGs and USMGs. For example, IMG surgical oncologists were more likely to care for Medicare beneficiaries with higher baseline risk scores versus USMGs; in turn, IMGs likely had different referral patterns with higher case mix indices, which also may have contributed to higher charges and reimbursement. Moreover, IMGs had more variation in the surgical procedures performed. In turn, the top‐paying services (fourth quartile) comprised a greater proportion of all services performed by IMGs (39.3% vs. 32.6%; *p* < 0.001) compared with USMGs. In particular, IMG surgical oncologists submitted a higher proportion of payments related to procedures, while USMG surgical oncologists submitted reimbursement for more E&M rather than procedural codes. Collectively, the data suggested that IMGs surgical oncologists provided a wider variety of services to a sicker patient population and, therefore, submitted higher charges per service and received higher payments per service.

Interestingly, there were also sex‐based variations and disparities in practice patterns and financial reimbursements among IMGs versus USMGs. There were very few female surgeons, especially IMG female surgical oncologists (*n* = 19, 18.6%), who treated Medicare beneficiaries. While the proportion of female IMG surgical oncologists did increase from 17% in 1981 to 26% in 2001, there remained a marked lack of female representation among IMG physicians.^30^ Lack of female surgical role models and lifestyle considerations have been proposed as reasons related to under‐representation of female surgeons.^31^, ^32^, ^33^ There are additional challenges faced by IMGs such as visa issues, sex‐based cultural expectations, and biases related to sex/gender, which could further account for the low female representation among IMGs.^9^, ^11^, ^27^, ^28^ In addition to low representation, female surgeons also had the lowest mean annual charges and payments, especially among female IMG surgical oncologists. These differences were not due to temporal or geographical variations, as the analysis was restricted to a single Medicare data file for 2021, and the CMS adjusted payment amounts were based on regional differences in the costs for medical services. As such, the data highlight persistent sex‐based payment disparities, especially among female IMGs providing surgical services. Sex‐ or gender‐based disparities in payments have been described for various other specialties including primary care, radiation oncology, cardiology, ophthalmology, and otolaryngology.^34^, ^35^, ^36^, ^37^, ^38^, ^39^, ^40^ For example, a study by Moore et al. reported lower compensation for female plastic surgeons compared with their male colleagues, shedding light on the payment inequity in surgical practice.^41^ Munir et al. also reported a similar trend in Medicare reimbursements across different surgical subspecialties.^20^ Payment‐based disparities likely were related, in part, to the lower prevalence of complex procedural codes and overall clinical volume among female surgeons.^42^ In the current study, female surgical oncologists performed fewer total services with a lower number of unique procedure codes. Such variations in female IMG surgical oncologist practice patterns may reflect disparities in referral practices. Importantly, female surgeons have been consistently demonstrated to have comparable or better clinical and surgical outcomes than their male counterparts.^43^, ^44^

IMG surgeons represent an important workforce to provide cancer patients with needed services, especially in healthcare provider shortage areas.^45^, ^46^, ^47^ Diversification of the workforce may also improve provisions of culturally sensitive care to diverse patient populations in the US, as well as increase access to care among patients residing in provider shortage areas.^48^, ^49^ Unfortunately, persistent legal and discriminatory obstacles hinder many IMGs from entering the US healthcare system, as well as hinder IMGs training as surgical oncologists in the US To address the shortage of healthcare providers, policymakers should ease entry barriers for IMGs by addressing visa restrictions, limited training opportunities, as well as addressing implicit bias.^2^, ^50^, ^51^ Understanding variations in practice patterns and payments among IMGs and USMGs not only demonstrates current disparities but also provides critical data that can guide healthcare administrators and policymakers in workforce planning.^20^, ^52^, ^53^ Specifically, this information can be utilized to strategically allocate medical resources and tailor recruitment efforts to ensure that areas with greater healthcare needs are staffed with appropriately skilled surgical oncologists. Resource allocation can be optimized to support the infrastructure needed for complex surgical care in underserved areas, ultimately improving healthcare access and equity. Additionally, targeted efforts are required to reduce gender‐based disparities in Medicare reimbursements and to increase the representation of female surgeons in the field.^17^, ^20^ As noted in the current study, IMG surgical oncologists provide critical oncologic surgical services, performing more procedural services and care for patients with a higher average risk scores. In addition, IMG surgical oncologists perform a greater variety of procedures compared with USMGs. Employment of IMGs to address healthcare worker shortages can help deliver essential cancer services in underserved areas and enhance overall cancer care.

Certain limitations should be considered while interpreting the results of the current study. We could only account for the medical school of graduation and could not differentiate between US versus non‐US‐IMGs. This information was not available due to data limitations. The lack of distinction may have limited the conclusions drawn about reimbursement differences, as differences in immigration and employment conditions may have influenced practice patterns. Furthermore, we could not differentiate between other subgroups, such as IMG surgeons who undertook alternate training and board certification routes. Furthermore, the inability to account for practice setting (academic vs. private practice) was another limitation that did not allow for reimbursement patterns among the surgeons practicing in these different clinical settings to be assessed. The use of a large administrative dataset may have lacked detailed information on factors like employment status, practice setting, and specific physician tasks. In turn, these limitations did not allow for the analysis of how these factors might affect Medicare‐related expenses and reimbursements. However, using the Medicare database permitted an assessment of billing variations within a structured dataset with standardized pricing. As this study was based on Medicare beneficiaries, the data may not be generalizable to surgeons who treated patients insured from private payers.

In conclusion, IMGs provided more procedural services, cared for patients with a higher average risk score, and performed a greater variety of procedures compared with USMGs. Consequently, IMGs had higher mean annual charges, payment‐per‐service, and charge‐per‐service amounts. The higher procedural burden and complexity of cases being managed by IMGs highlights the need for the healthcare system to strategically utilize and support this workforce to address healthcare shortages in specialized fields. Overall cancer care may be enhanced with the inclusion of IMGs to address healthcare worker shortages and deliver essential cancer services, particularly for managing more complex cases.

## AUTHOR CONTRIBUTIONS

**Muhammad Muntazir Mehdi khan**: Conceptualization; Data curation; Formal analysis; Investigation; Methodology; Visualization; Writing ‐ original draft; Writing ‐ review & editing. **Abdullah Altaf**: Conceptualization; Formal analysis; Methodology; Writing ‐ original draft; Writing ‐ review & editing. **Mujtaba Khalil**: Conceptualization; Methodology; Writing ‐ review & editing. **Sidharth Iyer**: Methodology; Writing ‐ original draft; Writing ‐ review & editing. **Razeen Thammachack**: Methodology; Writing ‐ original draft; Writing ‐ review & editing. **Abdul Hadi Shahid**: Conceptualization; Data curation; Methodology; Writing ‐ original draft; Writing ‐ review & editing. **Zayed Rashid**: Conceptualization; Data curation; Methodology; Writing ‐ review & editing. **Timothy M. Pawlik**: Conceptualization; Methodology; Supervision; Writing ‐ review & editing.

## CONFLICT OF INTEREST STATEMENT

The authors declare no conflicts of interest.

## ETHICS STATEMENT

This study was exempt from the institutional review board (IRB) approval as it involved the use of publicly available, de‐identified data from the Centers for Medicare & Medicaid Services (CMS) Medicare database. Since no direct interaction with human subjects occurred and no identifiable private information was used, the study was classified as nonhuman subjects' research. All data were handled in compliance with applicable data use agreements to ensure confidentiality and integrity.

## Supporting information

Supplementary Material

## Data Availability

The data for this study were obtained from the Centers for Medicare & CMS (CMS) Medicare fee‐for‐service provider utilization and payment files and are publicly available.

## References

[1] How IMGs have changed the face of American medicine [Internet]. American Medical Association. 2021 [cited 2023 Nov 27]. Available from: https://www.ama‐assn.org/education/international‐medical‐education/how‐imgs‐have‐changed‐face‐american‐medicine

[2] Abu Rous, Fawzi , and Ivy Riano . 2023 Mar 16. “Challenges Faced by J‐1 International Medical Graduates (IMGs) during COVID‐19 Pandemic.” Cancer Investigation 41(3): 249–252. 10.1080/07357907.2023.2177859. 36745487

[3] Chen, Peggy G.‐Chi , Marcella Nunez‐Smith , Susannah May Bernheim , David Berg , Aysegul Gozu , and Leslie Ann Curry . 2010 Sep 1. “Professional Experiences of International Medical Graduates Practicing Primary Care in the United States.” Journal of General Internal Medicine 25(9): 947–953. 10.1007/s11606-010-1401-2. 20502974 PMC2917670

[4] Carethers, John M. , Sandra M. Quezada , Rotonya M. Carr , and Lukejohn W. Day . 2019 Mar. “Diversity within US Gastroenterology Physician Practices: The Pipeline, Cultural Competencies, and Gastroenterology Societies Approaches.” Gastroenterology 156(4): 829–833. 10.1053/j.gastro.2018.10.056. 30452917 PMC6453700

[5] David, Yakira N. , and Rachel B. Issaka . 2021 Dec 1. “Advancing Diversity: the Role of International Medical Graduates.” Lancet Gastroenterol Hepatol 6(12): 980–981. 10.1016/s2468-1253(21)00376-9. 34774151 PMC8813365

[6] Physician characteristics and distribution in the US [Internet]. [Chicago, IL]: AMA Press; 2006 398 p. [cited 2024 Jun 4]. Available from: http://archive.org/details/physiciancharact0000unse_m9e2

[7] Polsky, Daniel , Philip R. Kletke , Gregory D. Wozniak , and José J. Escarce . 2002 Aug. “Initial Practice Locations of International Medical Graduates.” Health Services Research 37(4): 907–928. 10.1034/j.1600-0560.2002.58.x. 12236390 PMC1464010

[8] State‐Level Projections of Supply and Demand for Primary Care Practitioners: 2013‐2025.

[9] Murillo, Zepeda C. , F. O. Alcalá Aguirre , E. M. Luna Landa , E. N. Reyes Güereque , G. P. Rodríguez García , and L. S. Diaz Montoya . 2022 Jul. “Challenges for International Medical Graduates in the US Graduate Medical Education and Health Care System Environment: A Narrative Review.” Cureus 14(7): e27351. 35910699 10.7759/cureus.27351 PMC9334519

[10] Chen, Peggy G.‐Chi , Leslie Ann Curry , Susannah May Bernheim , David Berg , Aysegul Gozu , and Marcella Nunez‐Smith . 2011 Nov. “Professional Challenges of Non‐U.S.‐Born International Medical Graduates and Recommendations for Support during Residency Training.” Academic Medicine 86(11): 1383–1388. 10.1097/acm.0b013e31823035e1. 21952056 PMC3257160

[11] Healey, Sunita Joann Rebecca , Kristy Fakes , and Balakrishnan R. Nair . 2023 Jul 12. “Inequitable Treatment as Perceived by International Medical Graduates (IMGs): a Scoping Review.” BMJ Open 13(7): e071992. 10.1136/bmjopen-2023-071992. PMC10347491 37438072

[12] Zaheer, Salman , Samuel D. Pimentel , Kristina D. Simmons , Lindsay E. Kuo , Jashodeep Datta , Noel Williams , Douglas L. Fraker , and Rachel R. Kelz . 2017 May. “Comparing International and United States Undergraduate Medical Education and Surgical Outcomes Using a Refined Balance Matching Methodology.” Annals of Surgery 265(5): 916–922. 10.1097/sla.0000000000001878. 27429031

[13] Tsugawa, Yusuke , Justin B. Dimick , Anupam B. Jena , Melinda Maggard‐Gibbons , Daniel M. Blumenthal , Nate Gross , and Ashish K. Jha . 2021 Dec 1. “Comparison of Patient Outcomes of Surgeons Who Are US versus International Medical Graduates.” Annals of Surgery 274(6): e1047–e1055. 10.1097/sla.0000000000003736. 31850990

[14] Tsugawa, Yusuke , Anupam B. Jena , E. John Orav , and Ashish K. Jha . 2017 Feb 2. “Quality of Care Delivered by General Internists in US Hospitals Who Graduated from Foreign versus US Medical Schools: Observational Study.” BMJ 356: j273. 10.1136/bmj.j273. 28153977 PMC5415101

[15] Howard, Daniel L. , Carol D. Bunch , Wilberforce O. Mundia , Thomas R. Konrad , Lloyd J. Edwards , M. Ahinee Amamoo , and Yhenneko Jallah . 2006 Dec. “Comparing United States versus International Medical School Graduate Physicians Who Serve African‐ American and White Elderly.” Health Services Research 41(6): 2155–2181. 10.1111/j.1475-6773.2006.00587.x. 17116114 PMC1955313

[16] Weeks, William B. , and Amy E. Wallace . 2008. “Rural‐urban Differences in Primary Care Physicians’ Practice Patterns, Characteristics, and Incomes.” J Rural Health Off J Am Rural Health Assoc Natl Rural Health Care Assoc. 24(2): 161–170. 10.1111/j.1748-0361.2008.00153.x. 18397451

[17] Oshinowo, Temitope O. , Michael S. Rallo , Clemens M. Schirmer , and Lola B. Chambless . 2024 Jan 1. “Gender Differences in Medicare Practice and Payments to Neurosurgeons.” JAMA Surg 159(1): 35–42. 10.1001/jamasurg.2023.4988. 37819669 PMC10568441

[18] McPherson, Emily , Lindsay Hedden , and Dean A. Regier . 2016 Sep 21. “Impact of Oncologist Payment Method on Health Care Outcomes, Costs, Quality: a Rapid Review.” Systematic Reviews 5(1): 160. 10.1186/s13643-016-0341-2. 27653974 PMC5031322

[19] Moazzam, Zorays , Selamawit Woldesenbet , Muhammad Musaab Munir , Laura Alaimo , Henrique Lima , Alina Ashraf , Yutaka Endo , and Timothy M. Pawlik . 2023 Nov 10. “Immigrant Doctors and Their Role in US Healthcare.” Journal of Gastrointestinal Surgery 27(12): 2724–2732: [cited 2023 Dec 3]; Available from. 10.1007/s11605-023-05878-4. 37950096

[20] Munir, Muhammad Musaab , Mary Dillhoff , Susan Tsai , Courtney Collins , Priya Dedhia , and Timothy M. Pawlik . 2024 Sep 1. “Gender‐Based Variations in Medicare Reimbursements Among Different Surgical Subspecialties.” JAMA Surg 159(9): 1060–1070. 10.1001/jamasurg.2024.2298. 39046733 PMC11270248

[21] Woods, Scott E. , Aaron Harju , Shoba Rao , Julie Koo , and Divya Kini . 2006 Dec 1. “Perceived Biases and Prejudices Experienced by International Medical Graduates in the US Post‐Graduate Medical Education System.” Medical Education Online 11(1): 4595. 10.3402/meo.v11i.4595. 28253800

[22] Zaidi, Zareen , Mantosh Dewan , and John Norcini . 2020 Dec. “International Medical Graduates: Promoting Equity and Belonging.” Academic Medicine 95(12S): S82–S87. 10.1097/acm.0000000000003694. 32889932

[23] Medicare Physician & Other Practitioners ‐ Centers for Medicare & Medicaid Services Data [Internet]. [cited 2024 Jan 27]. Available from: https://data.cms.gov/provider‐summary‐by‐type‐of‐service/medicare‐physician‐other‐practitioners

[24] National Downloadable File | Provider Data Catalog [Internet]. [cited 2024 Jan 27]. Available from: https://data.cms.gov/provider‐data/dataset/mj5m‐pzi6

[25] Medicare Physician & Other Practitioners ‐ by Provider Data Dictionary ‐ Centers for Medicare & Medicaid Services Data [Internet]. [cited 2024 Jan 27]. Available from: https://data.cms.gov/resources/medicare‐physician‐other‐practitioners‐by‐provider‐data‐dictionary

[26] Restructured BETOS Classification System | CMS Data [Internet]. [cited 2024 Oct 14]. Available from: https://data.cms.gov/provider‐summary‐by‐type‐of‐service/provider‐service‐classifications/restructured‐betos‐classification‐system

[27] Chen, Peggy G.‐Chi , Marcella Nunez‐Smith , Susannah May Bernheim , David Berg , Aysegul Gozu , and Leslie Ann Curry . 2010 Sep. “Professional Experiences of International Medical Graduates Practicing Primary Care in the United States.” Journal of General Internal Medicine 25(9): 947–953. 10.1007/s11606-010-1401-2. 20502974 PMC2917670

[28] Pilotto, Louis S. , Geraldine F. Duncan , and Jane Anderson‐Wurf . 2007. “Issues for Clinicians Training International Medical Graduates: a Systematic Review.” Medical Journal of Australia 187(4): 225–228. 10.5694/j.1326-5377.2007.tb01204.x. 17708725

[29] Glover, McKinley, IV , Nathaniel D. Mercaldo , Daniel M. Blumenthal , Timothy G. Ferris , and Jason H. Wasfy . 2019 Jul 1. “Differences in Medicare Beneficiary Risk Scores by Physician’s International Medical Graduate Status.” Journal of General Internal Medicine 34(7): 1110–1112. 10.1007/s11606-019-04830-0. 30737681 PMC6614298

[30] Hart, L. Gary , Susan M. Skillman , Meredith Fordyce , Matthew Thompson , Amy Hagopian , and Thomas R. Konrad . 2007 Jul. “International Medical Graduate Physicians in the United States: Changes since 1981.” Health Affairs 26(4): 1159–1169. 10.1377/hlthaff.26.4.1159. 17630460

[31] Gargiulo, Debra A. , N. H. Hyman , and J. C. Hebert . 2006 Apr 1. “Women in Surgery: Do We Really Understand the Deterrents?” Archives of Surgery 141(4): 405–408. 10.1001/archsurg.141.4.405. 16618901

[32] Richardson, H. C. , and N. Redfern . 2000 Oct 1. “Why Do Women Reject Surgical Careers.” Annals of the Royal College of Surgeons of England 82(9 Suppl): 290–293. 11089452

[33] Sanfey, Hilary A. , A. R. Saalwachter‐Schulman , J. M. Nyhof‐Young , B. Eidelson , and B. D. Mann . 2006 Nov 1. “Influences on Medical Student Career Choice: Gender or Generation?” Archives of Surgery 141(11): 1086–1094. 10.1001/archsurg.141.11.1086. 17116801

[34] Seabury, Seth A. , Amitabh Chandra , and Anupam B. Jena . 2013 Oct 14. “Trends in the Earnings of Male and Female Health Care Professionals in the United States, 1987 to 2010.” JAMA Internal Medicine 173(18): 1748–1750. 10.1001/jamainternmed.2013.8519. 23999898

[35] Jagsi, Reshma , Kent A. Griffith , Abigail Stewart , Dana Sambuco , Rochelle DeCastro , and Peter A. Ubel . 2013 Nov. “Gender Differences in Salary in a Recent Cohort of Early‐Career Physician‐Researchers.” Acad Med J Assoc Am Med Coll 88(11): 1689–1699. 10.1097/acm.0b013e3182a71519. PMC3816636 24072109

[36] Valle, Luca , Julius Weng , Reshma Jagsi , F.‐I. Chu , Sumayya Ahmad , Michael Steinberg , and Ann Raldow . 2019 Mar 1. “Assessment of Differences in Clinical Activity and Medicare Payments Among Female and Male Radiation Oncologists.” JAMA Network Open 2(3): e190932. 10.1001/jamanetworkopen.2019.0932. 30901047 PMC6583310

[37] Reddy, Ashvini K. , Gregory W. Bounds , Sophie J. Bakri , Lynn K. Gordon , Justine R. Smith , Julia A. Haller , Audina M. Berrocal , and Jennifer E. Thorne . 2017 Mar 1. “Differences in Clinical Activity and Medicare Payments for Female vs Male Ophthalmologists.” JAMA Ophthalmol 135(3): 205–213. 10.1001/jamaophthalmol.2016.5399. 28114631

[38] Miller, Ashley L. , Vinay K. Rathi , Ciersten A. Burks , Elliana Kirsh DeVore , Regan W. Bergmark , and Stacey T. Gray . 2020 Sep 1. “Assessment of Gender Differences in Clinical Productivity and Medicare Payments Among Otolaryngologists in 2017.” JAMA Otolaryngol‐‐ Head Neck Surg. 146(9): 1–10. 10.1001/jamaoto.2020.1928. 32745204 PMC7393586

[39] Raber, Inbar , Mahmoud Al Rifai , Cian P. McCarthy , Muthiah Vaduganathan , Erin D. Michos , Malissa J. Wood , Yvonne M. Smyth , et al. 2021 Dec 1. “Gender Differences in Medicare Payments Among Cardiologists.” JAMA Cardiol 6(12): 1432–1439. 10.1001/jamacardio.2021.3385. 34495296 PMC8427494

[40] Behmer Hansen, Rosemary T. , Nicole A. Silva , Rebecca Cuevas , Samantha Y. Cerasiello , Angela M. Richardson , Antonios Mammis , and Anil Nanda . 2020 Aug 28. “Fellowship, Gender, and Scholarly Productivity: Trends Among Academic Neurosurgeons in the US.” Journal of Neurosurgery 135(1): 185–193. 10.3171/2020.5.jns20577. 32858514

[41] Moore, Meredith G. , Kyle W. Singerman , William J. Kitzmiller , and Ryan M. Gobble . 2021 Nov 1. “Gender Disparity in 2013‐2018 Industry Payments to Plastic Surgeons.” Aesthetic Surgery Journal 41(11): 1316–1320. 10.1093/asj/sjaa367. 33326584

[42] Dossa, Fahima , Dan Zeltzer , Rinku Sutradhar , Andrea N. Simpson , and Nancy N. Baxter . 2022 Feb 1. “Sex Differences in the Pattern of Patient Referrals to Male and Female Surgeons.” JAMA Surg 157(2): 95–103. 10.1001/jamasurg.2021.5784. 34757424 PMC8581775

[43] Tsugawa, Yusuke , Anupam B. Jena , Jose F. Figueroa , E. John Orav , Daniel M. Blumenthal , and Ashish K. Jha . 2017 Feb 1. “Comparison of Hospital Mortality and Readmission Rates for Medicare Patients Treated by Male vs Female Physicians.” JAMA Internal Medicine 177(2): 206–213. 10.1001/jamainternmed.2016.7875. 27992617 PMC5558155

[44] Wallis, Christopher J. D. , Bheeshma Ravi , Natalie Coburn , Robert K. Nam , Allan S. Detsky , and Raj Satkunasivam . 2017 Oct 10. “Comparison of Postoperative Outcomes Among Patients Treated by Male and Female Surgeons: a Population Based Matched Cohort Study.” BMJ 359: j4366. 10.1136/bmj.j4366. 29018008 PMC6284261

[45] Polsky, Daniel , Philip R. Kletke , Gregory D. Wozniak , and José J. Escarce . 2002. “Initial Practice Locations of International Medical Graduates.” Health Services Research 37(4): 907–928. 10.1034/j.1600-0560.2002.58.x. 12236390 PMC1464010

[46] Fitzsimons, M. G. , and B. M. C. de Oliveira . 2021. “The Role of International Medical Graduates (IMGs) in the US Healthcare System.” In International Medical Graduates in the United States: A Complete Guide to Challenges and Solutions [Internet], edited by H. Tohid and H. Maibach , 227–244. Cham: Springer International Publishing: [cited 2023 Dec 3]. 10.1007/978-3-030-62249-7_15.

[47] Tohid, H. and H. Maibach . 2021. International Medical Graduates in the United States: A Complete Guide to Challenges and Solutions [Internet]. Cham: Springer International Publishing: [cited 2023 Dec 31]. Available from: https://link.springer.com/10.1007/978‐3‐030‐62249‐7.

[48] Wilbur, Kirsten , Cyndy Snyder , Alison C. Essary , Swapna Reddy , Kristen K. Will , and Saxon S. Mary . 2020 Jun. “Developing Workforce Diversity in the Health Professions: A Social Justice Perspective.” Health Prof Educ 6(2): 222–229. 10.1016/j.hpe.2020.01.002.

[49] Mensah, Michael O. , and Benjamin D. Sommers . 2016 Nov. “The Policy Argument for Healthcare Workforce Diversity.” Journal of General Internal Medicine 31(11): 1369–1372. 10.1007/s11606-016-3784-1. 27431386 PMC5071285

[50] Kaafarani, Haytham M. A. 2009 Jul 1. “International Medical Graduates in Surgery: Facing Challenges and Breaking Stereotypes” Americas Journal of Surgery 198(1): 153–154. 10.1016/j.amjsurg.2008.08.004. 19095214

[51] Cohen, Jordan J. 2006 Dec. “The Role and Contributions of IMGs: A U.S. Perspective” Academic Medicine 81(12): S17–S21. 10.1097/01.acm.0000243339.63320.98. 17086040

[52] Heneghan, Steven J. , James Bordley, IV , Patrick A. Dietz , Michael S. Gold , Paul L. Jenkins , and Randall J. Zuckerman . 2005 Nov. “Comparison of Urban and Rural General Surgeons: Motivations for Practice Location, Practice Patterns, and Education Requirements.” Journal of the American College of Surgeons 201(5): 732–736. 10.1016/j.jamcollsurg.2005.06.262. 16256916

[53] Hagopian, Amy , Matthew J. Thompson , Emily Kaltenbach , and L. Gary Hart . 2004. “The Role of International Medical Graduates in America’s Small Rural Critical Access Hospitals.” The Journal of Rural Health 20(1): 52–58. 10.1111/j.1748-0361.2004.tb00007.x. 14974436

