# Supplementary material for: Variations in medicare reimbursements among surgical oncologists who are US versus international medical graduates
Source: World J Surg. 2024 Dec 22;49(2):512–22. doi: 10.1002/wjs.12458 (PMC11798682; doi:10.1002/wjs.12458)
Supplement: Supplementary file 1 — Supplementary Material [file WJS-49-512-s001.docx]

**Supplementary Table 1.** Variation in Payments and Charges within Matched Procedures among IMG vs USMG Surgeons.

|  | **USMG  Mean (SE), $** | **IMG Mean (SE), $** | **Difference, $^a^** |
| --- | --- | --- | --- |
| **Total Procedures** |  |  |  |
| Charges | 2188.69 | 1955.45 | 233.24 |
| Payments | 363.23 | 331.46 | 31.78 |
| Standardized Payments |  |  |  |
| **CPT codes (name), greatest varying^b^** |  |  |  |
| Ultrasound guidance for tissue destruction | 86.77 | 2453.16 | -2366.39 |
| Partial removal of pancreas, bile duct, and small bowel with connection of pancreas to small bowel | 2491.734 | 366.0969 | 2125.637 |
| Fluoroscopic guidance for insertion, replacement, or removal of central venous access device | 14.34 | 2086.961 | -2072.62 |
| Removal or destruction of (greater than 10.0 centimeters) abdominal cavity growths, cysts, or abnormal tissue, open procedure | 1319.945 | 326.6618 | 993.2828 |
| Removal of lymph nodes, muscle, and tissue of neck | 1360.774 | 220.1726 | 1140.601 |
| Partial removal of the pancreas | 1371.386 | 219.85 | 1151.536 |
| Partial removal of liver lobe | 1937.52 | 209.8425 | 1727.677 |
| Pancreas procedure | 1946.191 | 189.6883 | 1756.503 |
| Fine needle aspiration of additional lesion using ultrasound guidance | 42.36 | 914.6385 | -872.279 |
| Fluoroscopic guidance for insertion, replacement, or removal of central venous access device | 17.11 | 876.2173 | -859.107 |
| Removal of peripheral venous catheter for infusion | 183.87 | 1037.55 | -853.68 |
| Removal of malignant growth (over 4.0 centimeters) of the trunk, arms, or legs | 180.145 | 1016.714 | -836.569 |
| Biopsy or removal of lymph nodes of under the arm, open procedure | 158.04 | 984.105 | -826.065 |

^Abbreviations: CPT, Current Procedural Terminology.
a Differences in submitted charges and payments after matching all CPT codes.
b Top ten services with the largest payment discrepancy between IMG and USMG surgeons.^

**Supplementary Table 2.** Medicare Charges, Payments, Practice Volume, and Beneficiary Metrics among USMGs vs. IMGs stratified by surgeon sex.

|  | Female IMGs | Male IMGs | Female USMGs | Male USMGs | All | P-value |
| --- | --- | --- | --- | --- | --- | --- |
|  | (N=19) | (N=83) | (N=385) | (N=465) | (N=952) |  |
| Charges and payments, mean (SE) |  |  |  |  |  |  |
| Total submitted charges,  $ | 295,383.246 (200,412.418) | 424,407.123 (364,603.114) | 294,167.685 (517,405.832) | 414,543.420 (523,117.846) | 364,343.825 (507,417.230) | 0.004 |
| Total payments, $ | 57798.53 7,194.833 | 82498.83 6,440.811 | 61392.41 4,420.417 | 81936.55 3,075.641 | 73195.54 2,426.517 | <0.001 |
| Total standardized  payments, $^a^ | 55102.22 6,885.196 | 77617.14 5,973.709 | 58810.63 4,288.577 | 77433.45 2,809.406 | 69472.49 2,293.854 | <0.001 |
| Payment-to-charge ratio | 0.22 0.020 | 0.23 0.010 | 0.24 0.005 | 0.23 0.004 | 0.23 0.003 | 0.493 |
| Charge per service | 1308.41 262.415 | 1227.39 83.594 | 902.14 28.995 | 1108.24 40.819 | 1039.27 25.062 | <0.001 |
| Payment per service | 231.38 27.850 | 237.74 11.081 | 179.16 3.062 | 210.31 4.023 | 200.52 2.645 | <0.001 |
| Beneficiary characteristics, median (IQR) |  |  |  |  |  |  |
| Average Age, mean (SE) | 72.00 0.382 | 72.58 0.253 | 72.26 0.099 | 72.78 0.099 | 72.54 0.067 | 0.002 |
| Male, No. | 26.0 (16.0-36.0) | 52.0 (36.0-75.0) | 23.0 (0.0-53.0) | 58.0 (34.0-90.0) | 49.0 (24.0-80.0) | <0.001 |
| Female, No. | 69.0 (35.0-108.0) | 62.0 (41.0-94.0) | 63.5 (45.0-104.0) | 64.0 (43.0-105.0) | 64.0 (43.0-104.0) | 0.237 |
| Non-Hispanic White, No. | 89.5 (50.0-139.0) | 93.0 (68.0-161.0) | 106.5 (61.5-169.0) | 118.5 (74.0-196.5) | 110.0 (68.0-178.0) | 0.014 |
| Racial/ethnic minority,  No. | 42.0 (29.0-45.0) | 26.0 (15.0-39.0) | 25.0 (15.0-37.0) | 26.0 (15.0-44.0) | 26.0 (15.0-42.0) | 0.793 |
| Dual Medicare/Medicaid  eligibility, No. | 21.0 (16.0-29.0) | 25.0 (16.0-34.0) | 20.0 (14.0-28.0) | 21.0 (15.0-31.0) | 21.0 (15.0-30.0) | 0.027 |
| Average risk score, mean  (SE)^b^ | 1.33 0.087 | 1.78 0.039 | 1.24 0.019 | 1.64 0.020 | 1.48 0.015 | <0.001 |
| Practice volume, median  (IQR) |  |  |  |  |  |  |
| Total services, No. | 264.0 (171.0-357.0) | 311.0 (178.0-468.0) | 267.0 (171.0-415.0) | 323.0 (191.0-549.0) | 294.5 (182.0-488.5) | 0.004 |
| Total beneficiaries, No. | 92.0 (50.0-161.0) | 119.0 (85.0-178.0) | 108.0 (72.0-170.0) | 128.0 (88.0-204.0) | 119.0 (80.5-186.5) | 0.002 |
| Unique codes, No. | 37.0 (20.0-53.0) | 54.0 (37.0-72.0) | 26.0 (19.0-39.0) | 50.0 (33.0-72.0) | 39.0 (24.0-61.0) | <0.001 |

^Statistics presented: mean (SE), median (interquartile range).^

^a Standardized payments indicates adjustment for geographic differences.
b Risk score indicates adjustment for different patient risk profiles.^
